# Olfactory neuronal cells as a promising tool to realize the “druggable genome” approach for drug discovery in neuropsychiatric disorders

**DOI:** 10.3389/fnins.2022.1081124

**Published:** 2023-03-10

**Authors:** Marina Mihaljevic, Max Lam, Carlos Ayala-Grosso, Finn Davis-Batt, David J. Schretlen, Koko Ishizuka, Kun Yang, Akira Sawa

**Affiliations:** ^1^Department of Neuroscience, Johns Hopkins University School of Medicine, Baltimore, MD, United States; ^2^IMH Neuropsychiatric Genomics Laboratory, Institute of Mental Health, Singapore, Singapore; ^3^Population and Global Health, LKC Medicine, Nanyang Technological University, Singapore, Singapore; ^4^Neurogenomic Biomarkers Laboratory, Zucker Hillside Hospital, Glen Oaks, NY, United States; ^5^Stanley Center for Psychiatric Research, Broad Institute of MIT and Harvard, Cambridge, MA, United States; ^6^Unit of Cellular Therapy, Centre of Experimental Medicine, Instituto Venezolano de Investigaciones Cientificas IVIC, Caracas, Venezuela; ^7^Department of Psychiatry, Johns Hopkins University School of Medicine, Baltimore, MD, United States; ^8^Department of Pharmacology, Johns Hopkins University School of Medicine, Baltimore, MD, United States; ^9^Department of Biomedical Engineering, Johns Hopkins University School of Medicine, Baltimore, MD, United States; ^10^Department of Genetic Medicine, Johns Hopkins University School of Medicine, Baltimore, MD, United States; ^11^Department of Mental Health, Johns Hopkins Bloomberg School of Public Health, Baltimore, MD, United States

**Keywords:** olfactory neuronal cells, cognition, nalas biopsy, psychosis, drug discovery, CLCN2

## Abstract

“Druggable genome” is a novel concept that emphasizes the importance of using the information of genome-wide genetic studies for drug discovery and development. Successful precedents of “druggable genome” have recently emerged for some disorders by combining genomic and gene expression profiles with medical and pharmacological knowledge. One of the key premises for the success is the good access to disease-relevant tissues from “living” patients in which we may observe molecular expression changes in association with symptomatic alteration. Thus, given brain biopsies are ethically and practically difficult, the application of the “druggable genome” approach is challenging for neuropsychiatric disorders. Here, to fill this gap, we propose the use of olfactory neuronal cells (ONCs) biopsied and established via nasal biopsy from living subjects. By using candidate genes that were proposed in a study in which genetic information, postmortem brain expression profiles, and pharmacological knowledge were considered for cognition in the general population, we addressed the utility of ONCs in the “druggable genome” approach by using the clinical and cell resources of an established psychosis cohort in our group. Through this pilot effort, we underscored the *chloride voltage-gated channel 2 (CLCN2)* gene as a possible druggable candidate for early-stage psychosis. The *CLCN2* gene expression was associated with verbal memory, but not with other dimensions in cognition, nor psychiatric manifestations (positive and negative symptoms). The association between this candidate molecule and verbal memory was also confirmed at the protein level. By using ONCs from living subjects, we now provide more specific information regarding molecular expression and clinical phenotypes. The use of ONCs also provides the opportunity of validating the relationship not only at the RNA level but also protein level, leading to the potential of functional assays in the future. Taken together, we now provide evidence that supports the utility of ONCs as a tool for the “druggable genome” approach in translational psychiatry.

## 1. Introduction

Cognitive deficits are one of the key domains of psychopathology in psychotic disorders, and are present throughout the illness course, affecting social and occupational functioning, and leading to poorer outcomes (Fett et al., 2011; Cowman et al., 2021). Nevertheless, currently approved antipsychotic drugs have modest or negligible effects on cognitive improvement (Meltzer and McGurk, 1999; Baldez et al., 2021). Thus, it is warranted that the discovery and development of new drugs that primarily target cognitive deficits are taken place in a mechanism-driven manner.

“Druggable genome” is a novel concept, emerged in the post-genomic era, that emphasizes the significance of using the information of genome-wide genetic studies for drug discovery and development (Hopkins and Groom, 2002). Many studies have supported that promising molecular candidates for drug discovery are underscored in genome-wide genetic studies in a wide range of diseases, including brain disorders (Lencz and Malhotra, 2015; Wain et al., 2017; Lempiainen et al., 2018; Gaziano et al., 2021; Hegvik et al., 2021; Storm et al., 2021; Valette et al., 2021; Chia et al., 2022). Successful precedents of “druggable genome” have recently emerged for some disorders by combining genomic and gene expression profiles with medical and pharmacological knowledge (Nabirotchkin et al., 2020). One of the key premises for the success is the good access to disease-relevant tissues from “living” patients in which molecular expression changes are associated with symptomatic alteration.

In addition to the studies for each specific disorder, some genome-wide association studies have targeted specific phenotypes, such as cognitive impairment. For example, a recent study used the currently largest dataset of general cognitive ability for a genome-wide association study (GWAS) in general populations (Lam et al., 2021). In this study, the final candidates were narrowed from the GWAS hits by examining postmortem gene expression databases in association with genetic variation and cognition, considering whether their encoded proteins may mediate pharmacological actions of currently available medicines by literature mining. As a result, the study finalized 16 candidate genes.

The study by Lam and colleagues (Lam et al., 2021) may be one of the first “druggable genome” efforts for brain conditions, but it also includes a potential limitation in expanding this approach in neuropsychiatric disorders. To step forward with the “druggable genome” approach, the expression of candidate molecules defined by this approach are expected to be different between patients and healthy controls (HCs) in disease-relevant tissues, and/or to be correlated with some clinical and cognitive symptoms in “living” patients. Different from other physical disorders, such as cancers in which the “druggable genome” approach has been effectively used, it is difficult for neuropsychiatric disorders to evaluate molecular expression changes in disease-relevant tissues (e.g., brain cells and neuronal cells) in association with symptomatic alteration in “living” patients. This validation is essential for further pharmacological assessment.

To overcome this dilemma, we hypothesized that olfactory neuronal cells (ONCs) biopsied and established via nasal biopsy from living subjects might be a useful tool to fill the gap. Several groups have established similar but slightly different protocols for preparing ONCs, but all these cells are cultured *in vitro* and can be stored until further use. These cells share molecular signatures relevant to neurons (Fan et al., 2013; Kano et al., 2013; English et al., 2015; Feron et al., 2016; Lavoie et al., 2017; Rhie et al., 2018; Sumitomo et al., 2018; Doostparast Torshizi et al., 2019; Evgrafov et al., 2020; Takayanagi et al., 2021; Tee and Mackay-Sim, 2021; Yang et al., 2021). There are advantages of using ONCs to fill the gap in the “druggable genome” approach: first, we can conduct symptomatic and cognitive assessment in parallel to nasal biopsy and molecular analysis of ONCs from the same subjects; second, at least by our own protocol, ONCs can be used for biochemical and pharmacological studies because of their homogeneity shown at the histochemical levels (Kano et al., 2013; Takayanagi et al., 2021).

In the present study, we tested our hypothesis and aimed to prove the utility of ONCs in expanding the “druggable genome” approach in neuropsychiatric disorders. To address this, we referred to the aforementioned report (Lam et al., 2021), examined the expressions of these candidate genes in ONCs, and looked for their correlations with clinical and cognitive manifestations in patients with early-stage psychosis. Through these efforts, we now highlight chloride voltage-gated channel 2 (*CLCN2*) as a promising target for a specific dimension of cognition in early-stage psychosis, further supporting the utility of ONCs as a tool for the “druggable genome” approach in translational psychiatry.

## 2. Subjects and methods

### 2.1. Study cohort

We studied 64 HCs and 56 patients with psychotic disorders within 5 years after onset, the majority of whom (52 subjects) within 2 years after onset. As described in recent publications that addressed different scientific questions (Kamath et al., 2018; Faria et al., 2019; Wang et al., 2019; Narita et al., 2021; Etyemez et al., 2022; Yang et al., 2022), diagnoses of psychotic disorders were confirmed using the Structural Clinical Interview for DSM-IV Patient Edition (SCID) (Williams et al., 1992). The spectrum of psychotic disorders included schizophrenia (26 patients), bipolar disorder with psychotic features (14 patients), schizoaffective disorder (seven patients), major depressive disorder with psychotic features (five patients), not otherwise specified psychotic disorder (two patients), and schizophreniform disorder (two patients). Exclusion criteria for all participants included a history of head trauma, nasal trauma, nasal surgery, neurologic disorder, cancer, viral infection, and a reported history of intellectual disability. Individuals who were pregnant or taking anti-inflammatory agents or experiencing any other psychiatric conditions were also excluded. In addition, participants with an estimated IQ below 70 on the Hopkins Adult Reading Test were excluded from this study (Schretlen et al., 2009). Patients who reported active substance abuse or produced a urine drug screen positive for illicit substance use, except cannabis, were excluded. This study was approved by the Johns Hopkins Medicine Institutional Review Boards and in accordance with The Code of Ethics of the World Medical Association (1964 Declaration of Helsinki). All study participants provided written informed consent. Parental consent and assent were obtained for all participants under the age of 18 years.

### 2.2. Symptomatic assessment and clinical data

The presence and severity of positive and negative symptoms of patients were evaluated by study physicians through the Scale for the Assessment of Negative Symptoms (SANS) and the Assessment of Positive Symptoms (SAPS) (Andreasen, 1990). Each symptom category includes a global rating score. The total global score, the sum of the global ratings, was used to calculate positive and negative symptoms.

The antipsychotic dosages were converted to chlorpromazine equivalents (CPZ) using the defined daily dose method (Leucht et al., 2016). Duration of illness (DOI) (between the onset and nasal biopsy), and CPZ doses were obtained through self-reports and confirmed through the Johns Hopkins electronic medical database. Note that we did not include data of cannabis use in the analysis, as the data were based on mere self-reports and they did not have quantitative value (answers were yes/no).

### 2.3. Neurocognitive assessment

We used a comprehensive neuropsychological battery which we previously developed and have used in our publications (Faria et al., 2019; Coughlin et al., 2021). We obtained cognitive scores scaled in normally distributed standardized units that covered six domains: (1) processing speed (calculated from the combined scores of the Grooved Pegboard test and the Salthouse test); (2) attention / working memory (Digit Span and Brief Attention Memory test); (3) verbal learning and memory (Hopkins Verbal Learning test); (4) visual learning and memory (Brief Visuospatial Memory test); (5) ideational fluency (Ideational Fluency assessment for Word Fluency and Acceptable Designs); and (6) executive functioning (Modified Wisconsin Card Sorting test). The median of the time gap between neurocognitive assessment and nasal biopsy for cell collection was 22 days.

### 2.4. Olfactory neuronal cells

The nasal biopsy was conducted at the Johns Hopkins Otolaryngology Clinic, and ONCs were prepared from nasal biopsied tissues obtained from patients and HCs in the Johns Hopkins Schizophrenia Center according to previous publications (Kano et al., 2013; Mor et al., 2013; Sumitomo et al., 2018; Takayanagi et al., 2021; Jaaro-Peled et al., 2022; Namkung et al., 2022). We collected 1-mm nasal biopsied tissues including olfactory neuroepithelium. The tissues were first incubated with 2.4 U/mL Dispase II for 45 min at 37°C, and then mechanically minced into small pieces. The tissue pieces were further treated with 0.25 mg/mL collagenase A for 10 min at 37°C. Dissociated cells were gently suspended and centrifuged to obtain pellets. Cell pellets were resuspended in D-MEM/F12 supplemented with 10% FBS and antibiotics (D-MEM/F12 medium), and tissue debris was removed by transferring only the cell suspension into a new tube. Cells were then plated on 6-well plates in fresh D-MEM/F12 medium (Day 0 plate). Cells floating were collected in fresh D-MEM/F12 medium on day 2 (Day 2 plate) and day 7 (Day 7 plate). Through these processes, connective tissue-origin cells and non-neuronal cells that are attached in Day 0 and 2 plates were removed. Cells in Day 7 plate were cultured until they reached confluency, collected, and stored in a liquid nitrogen tank until further use as ONCs. ONCs are nearly homogenous with a βIII-tubulin, a marker for immature neurons (Kano et al., 2013). Consistently, a genome-wide expression analysis of ONCs in reference to various pubic databases for gene expression also indicated that the general signature of ONCs is similar to that of developing neurons (Horiuchi et al., 2013).

### 2.5. RNA-sequencing

We have previously published the method for the RNA-sequencing study that was also employed in the present study (Jaaro-Peled et al., 2022). In brief, total RNA was isolated from the ONCs using the RNeasy Plus Mini Kit (Qiagen). RNA libraries were prepared from 500 ng total RNA using NEBNext Ultra II Directional RNA Library Prep Kit for Illumina (E7760 and E7490) following the NEBNext Poly(A) mRNA Magnetic Isolation Module protocol. Libraries were then normalized to 4 nM and pooled in equimolar amounts. Paired-End Sequencing was performed using Illumina's NovaSeq6000 S4 200 cycle kit.

FastQC (Wingett and Andrews, 2018) was used to check the quality of reads. High-quality data were obtained from raw data by using cutadapt (Martin, 2011) to remove adapters, primers, and reads with low quality (option -q 10) or shorter than 20 nt. Hisat2 was used to map the clean reads to the human genome (Pertea et al., 2016), version GRCh38 (Genome Reference Consortium Human Build 38). Stringtie (Pertea et al., 2016) was used to assemble and merge transcripts and estimate transcript abundances. A Python script (prepDE.py) provided by Stringtie developer was used to create count tables for further analysis.

### 2.6. CLCN2-encoded protein [chloride-channel 2 protein] expression levels

*ClC2* levels were measured in whole lysates of ONCs. ONCs obtained from 20 patients were lysed in RIPA buffer (50 mM Tris, pH 7.4, 150 mM NaCl, 1% NP-40, 0.5% sodium deoxycholate, and 0.1% SDS) containing proteinase inhibitor and phosphatase inhibitor cocktails and sonicated. Cell lysate samples (30 μg of protein) were run on NuPAGE 10% bis-tris gels (Invitrogen, NP0302BOX), transferred to PVDF membranes (Millipore, IPVH00010), and blocked for 1 h at room temperature in 5% milk in Tris buffered saline with 0.1% Tween 20. Membranes were incubated with an antibody against CLCN2 (Alomone Labs, ACL-002) (1:200 dilution) or with an antibody against GAPDH (Santa Cruz Biotech, sc-32233) (1:4000 dilution) overnight at 4°C, washed, and incubated in a horseradish peroxidase-conjugated secondary antibody (GE Healthcare, NA934V for CLCN2; NA931V for GAPDH) (1:5000 dilution) for 1 h at room temperature. After washing, membranes were developed using the Super Signal West Dura Chemiluminescent Substrate (Thermo Scientific). Protein band intensity was measured using ImageJ software (National Institutes of Health, Bethesda, MD). The ratio CLCN2/GAPDH was calculated and is plotted as mean ± SEM of independent samples.

### 2.7. Statistical analysis

SPSS v24.0 was used to perform statistical analysis. Group comparisons of demographic data were calculated using independent *t*-tests for continuous variables, and chi-squared tests for categorical variables. ANCOVA (analysis of covariance) controlling for age, sex, and race was used to compare the expressional levels of the gene candidates between HCs and patients. ANCOVA controlling for age, sex, race, and education was used to compare neurocognitive functions between HC s and patients. Linear regression with age, sex, and race as covariates was used to evaluate the correlation between the molecular expressions and clinical factors such as SANS/SAPS, CPZ, and DOI.

We conducted a one-tailed linear regression with age, sex, race, and the years of education as covariates to test whether the expression level of each candidate genes/protein is correlated with neurocognitive function in the same direction of correlation that was indicated in the study by Lam and colleagues (Lam et al., 2021). Bonferroni procedure is used for multiple comparison correction.

## 3. Results

### 3.1. Main characteristics of study participants

The demographic, clinical, and neurocognitive information for HCs and patients are summarized in Table 1 and Supplementary Table S1. There were differences in sex and the years of education, but not in age and race, between HCs and patients. All the demographic data were controlled in downstream analyses. There were differences in all neurocognitive domains, except IQ measures, between HCs and patients.

**Table 1:** Main characteristics of study participants.

	**Patients**	**HCs**	***p*-value**
**Demographic data**			
Age (years)	22.9 ± 3.9	23.3 ± 3.3	0.478
Sex (male)	48.4	69.6	**0.019**
Race (white)	35.7	37.5	0.840
Education (years)	13.0 ± 2.3	14.8 ± 1.9	**< 0.001**
**Clinical data**			
SAPS total score	15.5 ± 18.8	NA	
SANS total score	28.7 ± 21.9	NA	
Duration of illness	17.6 ± 13.5	NA	
CPZ dose	286.7 ± 237.8	NA	

Significant results are presented in bold.

### 3.2. Association of gene expression in ONCs with clinical and cognitive phenotypes

We first tested whether 16 gene candidates underscored by the published study (Lam et al., 2021) were expressed in ONCs. By examining our RNA-sequencing dataset, we confirmed that eight genes are expressed in ONCs. These included carbonic anhydrase 13 (*CA13*), chloride voltage-gated channel 2 (*CLCN2*), dihydroorotate dehydrogenase (*DHODH*), dipeptidyl peptidase 4 (*DPP4*), 5-hydroxytryptamine receptor 1D (*HTR1D*), phosphodiesterase 4D (*PDE4D*), proteasome 20S subunit alpha 5 (*PSMA5*), and thyroid hormone receptor beta (*THRB*). However, expression levels of these eight genes themselves were not different between patients and HCs in the group comparison (Table 2). This suggested that the impact of these genes, if exists, may be more specific to distinct phenotype(s), such as symptomatic and neurocognitive manifestations.

**Table 2:** Differences in gene expression levels between patients and HCs.

**Genes**	**Patients**	**HCs**	***p*-value**
CA13	0.48 ± 0.27	0.46 ± 0.27	0.702
CLCN2	1.09 ± 1.6	1.08 ± 0.19	0.226
DHODH	1.79 ± 0.21	1.79 ± 0.24	0.771
DPP4	1.01 ± 0.54	0.99 ± 0.60	0.826
HTR1D	0.23 ± 0.22	0.23 ± 0.16	0.698
PDE4D	0.89 ± 0.34	0.67 ± 0.35	0.500
PSMA5	4.62 ± 0.22	4.63 ± 0.23	0.837
THRB	1.43 ± 0.46	1.41 ± 0.46	0.864

Data are presented as mean ± SD. All analyses were controlled for age, sex, and race.

Accordingly, we decided to study the relationship between molecular expression and clinical phenotypes for these eight genes. First, we addressed whether the molecular expressions might be associated with symptomatic manifestations, such as positive and negative symptoms. We did not observe any associations between SAPS/SANS scores and molecular expression (Supplementary Table S2), indicating that the molecular expression and symptomatic manifestations are unrelated. We also tested whether CPZ doses and DOI were associated with the molecular expression and did not observe any associations (Supplementary Table S2). These results support the idea that medication and DOI (potential clinical confounders) do not influence the expression of these candidates.

Thus, we looked for possible associations between neurocognitive domains and molecular expressions. Our analysis highlighted 2 correlations: *CLCN2* expression with verbal memory in patients and *CLCN2* expression with executive function in HCs (Table 3).

**Table 3:** Association between verbal memory and executive function with gene expression levels.

	**Verbal memory**			**Executive function**		
	***t*** **-value**	***p*** **-value**	***q*** **-value**	***t*** **-value**	***p*** **-value**	***q*** **-value**
**Patients**						
CA13	−1.120	0.135	1.000	−0.965	0.170	1.000
CLCN2	2.819	**0.004**	**0.028**	0.171	0.433	1.000
DHODH	1.775	0.959	1.000	−1.085	0.142	1.000
DPP4	0.926	0.821	1.000	0.427	0.665	1.000
HTR1D	−1.985	0.974	1.000	−1.293	0.899	1.000
PDE4D	−1.048	0.150	1.000	−0.458	0.325	1.000
PSMA5	1.248	0.891	1.000	−0.729	0.235	1.000
THRB	−1.024	0.845	1.000	0.337	0.369	1.000
**HCs**						
CA13	0.038	0.516	1.000	1.966	0.973	1.000
CLCN2	0.049	0.481	1.000	3.203	**0.001**	**0.008**
DHODH	0.071	0.528	1.000	2.622	0.995	1.000
DPP4	0.690	0.754	1.000	0.211	0.584	1.000
HTR1D	−0.811	0.790	1.000	−0.933	0.838	1.000
PDE4D	−1.179	0.122	0.972	−0.105	0.459	1.000
PSMA5	−0.313	0.378	1.000	0.186	0.574	1.000
THRB	−0.523	0.699	1.000	−0.647	0.740	1.000

All analyses were controlled for age, sex, race, and education. *q*-value (FDR). Significant results are presented in bold.

### 3.3. CLCN2 in patients

**Figure 1:**
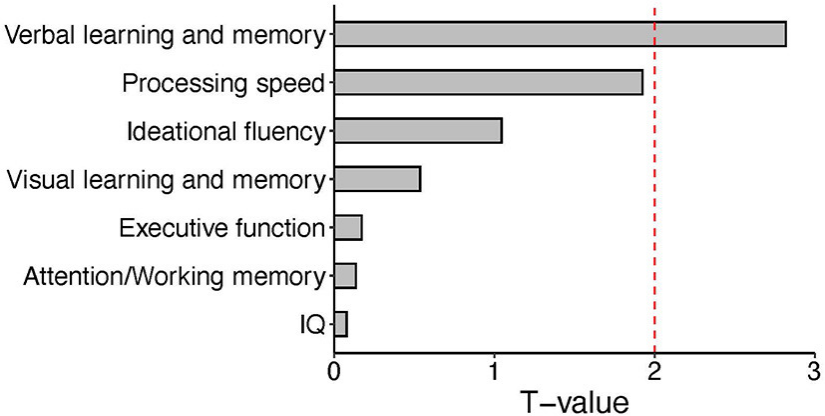
Bar plot of T values from the linear regression analysis between expression levels of the *CLCN2* gene and each neuropsychological domain. We tested the association between *CLCN2* expressional levels and all neuropsychological domains in patients. A significant correlation was observed for verbal memory. The red dashed line represents the significant cut-off (*p* < 0.05).

**Figure 2:**
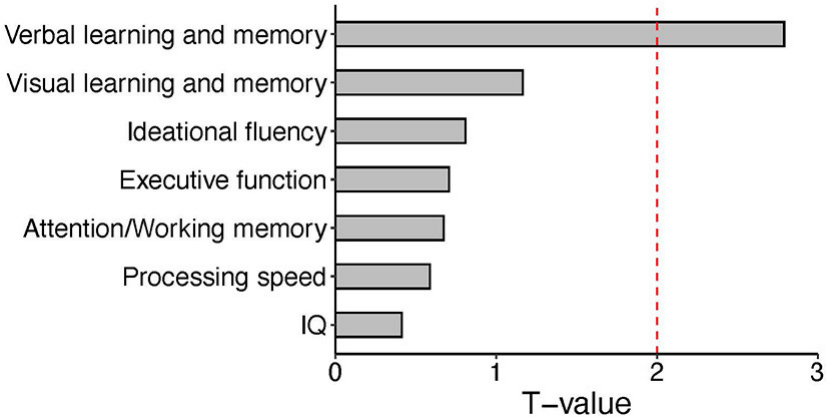
Bar plot of T values from the linear regression analysis between expression levels of the CLC-2 protein and each neuropsychological domain. We tested the association between CLC-2 and all neuropsychological domains in patients. A significant correlation was observed for verbal memory. The red dashed line represents the significant cut-off (*p* < 0.05).

As described above, expression of *CLCN2* at RNA levels (RNA-seq data) was positively associated with verbal memory in patients and executive function in HCs. Based on our perspectives of the “druggable genome” approach as described in the Introduction, we focused on the association between *CLCN2* gene expression and verbal memory in patients (Figure 1). Accordingly, we tried to validate the observations not only at the RNA levels but also at the protein levels. We examined the ClC-2 levels from patients and tested its association with neuropsychological domains. Results showed a strong association between ClC-2 level and verbal memory (Figure 2). The whole model explained around 60% of verbal memory variance (*R*^2^ = 0.617). The association had the same positive direction as that in the association between the *CLCN2* gene expression level and verbal memory. No association was found for any other neuropsychological domains.

## 4. Significance of the present study and future perspectives

### 4.1. The significance and high utility of ONCs in a “druggable genome” approach

The goal of the present study is to test the utility of ONCs in expanding the “druggable genome” approach in neuropsychiatric disorders from the information of genetic studies. We used the data from a report that combined the information of GWAS and postmortem expression study in association with general cognitive ability in the general population, as the initial information to explore the present study that includes molecular expression in ONCs. We examined the molecular expression with psychiatric manifestations (positive symptoms and negative symptoms) and 6 distinct cognitive domains in patients and HCs, and underscored *CLCN2* as a candidate for future drug discovery.

We regard that the study from which we obtained the initial information (Lam et al., 2021) is a respectful, pioneered effort of exploring the “druggable genome” approach in brain conditions. However, without the information on molecular expression and clinical manifestations from the same set of living subjects, the study was limited by the design of connecting genomic data with postmortem transcriptomics and cognitive data from different populations. In contrast, by studying the same subjects for ONC molecular expression and clinical manifestations, we could observe a correlation of *CLCN2* gene expression specific with verbal memory. We did not observe correlations with other dimensions in cognition, nor psychiatric manifestations (positive and negative symptoms). The association between this candidate molecule and verbal memory was also confirmed at the protein level. By using ONCs from living subjects, we now provide more specific information regarding molecular expression and clinical phenotypes, compared with the study we quoted for the initial resource. The use of ONCs also provides the opportunity of validating the relationship not only at the RNA level but also protein level, leading to the potential of functional assays in the future. Taken together, we now provide evidence that supports the utility of ONCs as a tool for the “druggable genome” approach in translational psychiatry.

### 4.2. Potential limitations of the present study

ONCs did not express all genes that were underscored in the study we used for the initial resource (Lam et al., 2021). This indicates that, although ONCs are useful as described above, we may not be able to validate all genes for the “druggable genome” approach through this experimental system. We acknowledge that this is a limitation in the use of ONCs.

As described in the Introduction section, we defined the criteria of candidate genes in the present study to be different in their expression between patients and healthy controls (HCs), and/or to show a correlation of the expression with clinical and cognitive symptoms in patients. We have to admit that the *CLCN2* gene satisfied the second criterion but not the first one. We observed a correlation between the *CLCN2* gene expression and executive function in HCs, but not in patients. Transparently speaking, we have no way of reasonably interpreting the data at least at present. However, although we focus our target only on patients based for the goal of “druggable genome” in the present study, we acknowledge that this is an interesting topic in future studies.

Furthermore, sense of smell is an important feature of the olfactory system, which include many factors and elements (cells in the OE, connectivity of the OE and olfactory blub, as well as neural network of OB, olfactory primary cortex, and higher cortex). However, we used ONCs as a surrogate neuronal tissue of analyzing gene expression level related to “druggable genes” for cognition. Exploring a potential role of ONCs in smell is a separate scientific question, which is an important future topic. Finally, we acknowledge that the sample size of the present cohort is relatively small. We hope that this pioneered study focusing on CLCN2 with a modest sample size, nevertheless clearly providing the utility of this “druggable genes” approach, may encourage future studies with cohorts of a larger sample size. If this is the case, the potential occurrence and possible implication of genetic variations in the *CLCN2* gene may be a future topic. Protein study with targets other than CLCN2/ClC2 will also be explored.

### 4.3. ClC-2 and lubiprostone

ClC-2 is a voltage-gated chloride channel that regulates chloride homeostasis in multiple tissues, such as the gastric tissue, testis, and retina (Goppner et al., 2021; Hanke-Gogokhia et al., 2021). Although its role in the brain remains elusive, the ClC-2 deficiency has been associated with leukoencephalopathy that accompanies psychiatric manifestation and cognitive impairment (Depienne et al., 2013; Guo et al., 2019). Some genetic studies have reported a possible association between *CLCN2* variants and neurodevelopmental conditions such as autistic spectrum disorders and epilepsy (Kleefuss-Lie et al., 2009; Cukier et al., 2014). Recent GWAS have reported its association with cognitive traits (Lee et al., 2018; Davies et al., 2019; Demange et al., 2021). From the “druggable genome” perspective, we note that Lubiprostone, a prostaglandin metabolite prostone, can activate ClC-2 Cl^−^ currents and Cl^−^ transport with an EC_50_ of ~18–24 nM in a protein kinase A-independent manner (Cuppoletti et al., 2004, 2014). Oral administration of Lubiprostone is effective to the treatment of constipation and irritable bowel in clinical settings specifically targeting CIC-2. Meanwhile, the trial of this medicine for patients with neuropsychiatric disorders was made only for the management of constipation (Pedrosa Carrasco et al., 2018; Xu et al., 2021). In contrast, our study has highlighted the potential implication of CIC-2 (*CLCN2*-encoded protein) in the context of a specific domain of cognition. Repurposing Lubiprostone in cognitive and neuropsychiatric disorders may be a promising strategy. If this is the case, this will be a proof of concept for the “druggable genome” approach.

## Data availability statement

Datasets are available on request: The raw data supporting the conclusions of this article will be available upon requests to the authors.

## Ethics statement

The studies involving human participants were reviewed and approved by Johns Hopkins Institute Review Board. Written informed consent to participate in this study was provided by the participants' legal guardian/next of kin.

## Author contributions

The current research was designed by AS and MM. The analytic pipeline was designed by KY, ML, and MM. The data was analyzed by MM, ML, and FD-B. Data analysis and interpretation regarding clinical scales were assisted by MM and DS. Data analysis and interpretation regarding molecular data were assisted by KI and CA-G. The manuscript was drafted by MM, KY, and AS. All authors contributed to the discussion of the results and have approved the final manuscript to be published.
