# Supplementary material for: Olfactory neuronal cells as a promising tool to realize the “druggable genome” approach for drug discovery in neuropsychiatric disorders
Source: Front Neurosci. 2023 Mar 10;16:1081124. doi: 10.3389/fnins.2022.1081124 (PMC10038100; doi:10.3389/fnins.2022.1081124)
Supplement: Supplementary file 1 [file Data_Sheet_1.PDF]

Supplementary Information

Olfactory neuronal cells as a promising tool to realize the "druggable genome" approach for drug discovery in neuropsychiatric disorders

Marina Mihaljevic[1], Max Lam[2,3,4,5], Carlos Ayala-Grosso[6], Finn Davis-Batt[7], David Schretlen[7], Koko Ishizuka[7], Kun Yang[7], and Akira Sawa[1,7,8,9,10,11*]

[1]Department of Neuroscience, Johns Hopkins University School of Medicine, Baltimore, MD,

[2]IMH Neuropsychiatric Genomics Laboratory, Institute of Mental Health, Singapore,

[3]Population and Global Health, LKC Medicine, Nanyang Technological University, Singapore,

[4]Neurogenomic Biomarkers Laboratory, Zucker Hillside Hospital, Glen Oaks, NY, USA,

[5]Stanley Center for Psychiatric Research, Broad Institute of MIT and Harvard, Cambridge, MA, USA,

[6]Unit of Cellular Therapy, Centre of Experimental Medicine, Instituto Venezolano de Investigaciones Cientificas IVIC, Caracas, Venezuela ,

Departments of [7]Psychiatry, [8]Pharmacology, [9]Biomedical Engineering, and [10]Genetic Medicine, Johns Hopkins University School of Medicine, Baltimore, MD.

[11]Department of Mental Health, Johns Hopkins Bloomberg School of Public Health, Baltimore, MD.

*Correspondence author

The running title: Olfactory neuronal cells in psychiatry

Akira Sawa: asawa1@jhmi.edu

Postal address: 600 N Wolfe St., Baltimore, MD 21287, USA

Main text: 3525 words

Figures: 2

Tables: 3

Table S1. Differences in NP domains between patients and HCs.

NP domains	Patients	HCs	p-value
IQ	98.17±8.82	104.37±10.79	*0.059*
Processing speed	98.98±11.46	112.79±8.89	**<0.001**
Attention/Working memory	88.47±12.74	103.64±11.03	**<0.001**
Verbal learning and memory	90.31±14.88	107.43±12.05	**<0.001**
Visual learning and memory	98.59±13.78	111.31±11.14	**0.001**
Ideational fluency	93.35±12.81	106.11±10.19	**<0.001**
Executive function	92.13±12.87	102.63±8.04	**0.001**

Note: Data are presented as mean±SD All analyses were controlled for age, sex, race, and education.

Table S2. Association between clinical phenotype and gene expression levels in patients

Genes	SAPS (q-value)	SANS (q-value)	CPZ doses (q-value)	DOI (q-value)
CA13	1.000	1.000	1.000	1.000
CLCN2	0.216	1.000	1.000	1.000
DHODH	0.075	1.000	1.000	1.000
DPP4	1.000	1.000	1.000	1.000
HTR1D	1.000	1.000	0.912	0.752
PDE4D	1.000	1.000	1.000	1.000
PSMA5	1.000	1.000	1.000	1.000
THRB	1.000	1.000	1.000	1.000

Note: All analyses were controlled for age, sex, and race.
